# The Molecular Mechanisms and Therapeutic Prospects of Alternative Lengthening of Telomeres (ALT)

**DOI:** 10.3390/cancers15071945

**Published:** 2023-03-23

**Authors:** Eric J. Sohn, Julia A. Goralsky, Jerry W. Shay, Jaewon Min

**Affiliations:** 1 Institute for Cancer Genetics, Columbia University Irving Medical Center, New York, NY 10032, USA; 2 Department of Cell Biology, University of Texas Southwestern Medical Center, Dallas, TX 75390-9039, USA; 3 Department of Pathology and Cell Biology, Columbia University Irving Medical Center, New York, NY 10032, USA

**Keywords:** cancer, review, ALT, telomere, homology directed repair, therapeutics

## Abstract

**Simple Summary:**

This review summarizes the current understanding of the telomere maintenance mechanism known as the Alternative Lengthening of Telomeres (ALT). The role, recognizable indicators, and proposed mechanism of the ALT pathway in sustaining cancer cells are reviewed. Potential molecular targets for future therapeutic development are proposed with the goal of synthesizing the current understanding of the ALT pathway that will be required to make future advances in ALT cancer treatments.

**Abstract:**

As detailed by the end replication problem, the linear ends of a cell’s chromosomes, known as telomeres, shorten with each successive round of replication until a cell enters into a state of growth arrest referred to as senescence. To maintain their immortal proliferation capacity, cancer cells must employ a telomere maintenance mechanism, such as telomerase activation or the Alternative Lengthening of Telomeres pathway (ALT). With only 10–15% of cancers utilizing the ALT mechanism, progress towards understanding its molecular components and associated hallmarks has only recently been made. This review analyzes the advances towards understanding the ALT pathway by: (1) detailing the mechanisms associated with engaging the ALT pathway as well as (2) identifying potential therapeutic targets of ALT that may lead to novel cancer therapeutic treatments. Collectively, these studies indicate that the ALT molecular mechanisms involve at least two distinct pathways induced by replication stress and damage at telomeres. We suggest exploiting tumor dependency on ALT is a promising field of study because it suggests new approaches to ALT-specific therapies for cancers with poorer prognosis. While substantial progress has been made in the ALT research field, additional progress will be required to realize these advances into clinical practices to treat ALT cancers and improve patient prognoses.

## 1. Introduction

### Telomere Maintenance Mechanisms

Telomeres are non-coding short repeat sequences (TTAGGG in vertebrates) which in combination with shelterin proteins protect the ends of linear chromosomes from degradation, recombination, and end fusions [1]. Human telomeres range from 5–15 kb in length [2]. Each cell culture replication cycle results in the loss of 50–200 bps due to incomplete end-replication and other telomere processing events [3]. During semi-conservative replication, the parent chromosomes serve as templates for the daughter chromosomes. The leading strand is synthesized continuously 5′ to 3′ by DNA polymerase while the lagging strand requires RNA primers to replicate the template strand. This primer does not start at the end of the chromosome which results in the shortening of the replicated chromosome [3]. The chromosome ends are further shortened by telomere end-processing to form 3′-overhangs. Exposed double-stranded telomere ends trigger DNA damage repair (DDR) pathways in the G2 cell cycle phase. To protect against DDR, telomere ends form t-loops in which a 3′ overhang invades and hybridizes with the proximal strand. Dimeric TRF1 (telomere-repeat binding factor 1), TRF2, and POT1 (protection of telomeres protein 1) are telomere binding proteins which recognize TTAGGG sequences and recruit TIN2 (TRF1-interacting nuclear factor 2), TPP1, and RAP1 [4,5]. Together these six proteins form the shelterin complex. The multiprotein shelterin complex recruits the APOLLO exonuclease which resects the 5′ telomere end. The resulting guanine rich 100–300 bp 3′ overhang forms a lariat structure known as the t-loop [3,6,7]. When a subset of telomeres are critically short, the protective shelterin complex is disrupted. Replicative senescence is considered an initial tumor suppression mechanism that is triggered by shortened telomeres. Genome instability such as fusion between chromosomes and double stranded breaks (DSBs) trigger senescence, a process of arrested cell growth, by activating the tumor suppressors including p53 and Rb [8,9,10].

One hallmark of neoplastic transformed cells is replicative immortality via telomere maintenance mechanisms. STEM cells and most tumor cells express telomerase, a reverse transcriptase which synthesizes new telomeric tandem arrays, and is repressed in almost all normal somatic cells [11]. The human Telomerase Reverse Transcriptase (hTERT) interacts with TPP1 to bind to telomeric DNA during the S and late G2 cell cycle phases [12,13,14,15,16,17]. Tumor cells with extended telomeric regions can then evade the telomere end problem and suppress DDR. However, approximately 10–15% of all cancers exhibit Alternative Lengthening of Telomeres (ALT), a telomerase-independent mechanism for lengthening telomeres by a DNA recombination mechanism. ALT cancers exhibit a wide variety of aberrant telomeric maintenance mechanisms involving Homology Directed Repair (HDR). Genomic instability gives rise to telomere fragments which accumulate in PML bodies. The telomere ends are extended by recombination using a telomere template sequence. Human ALT cancers are often present as mesenchymal or epithelial origin in subsets of osteosarcomas, liposarcomas, glioblastomas, or astrocytomas [18,19,20,21,22,23,24,25].

The study of ALT is currently limited by a lack of standardization. There does not exist one universal marker for ALT activity; instead, many studies choose to investigate 1–2 hallmarks such as APBs, c-circles, or heterogenous telomere length to establish ALT. As a result, a wide variety of tumors fall into the ALT category. We believe defining ALT biomarkers is an important goal for future studies and clinical application as ALT status conveys complex prognostic information. Studies of patient tissues show that ALT positive status indicates poor prognosis such as in neuroblastomas, osteosarcomas, and liposarcomas [21,26,27,28,29,30]. However, ALT positive glioblastoma portends better survival [19,20,31]. Future in vitro investigations into novel ALT models may elucidate the relationship and provide a more useful cancer prognostic tool.

In rare cases, ALT is exhibited upon telomerase inhibition. In vitro TERC knockout in telomerase positive H1299 and SW39 cells resulted in ALT pathway engagement in a very low frequency. Additionally, inhibition of telomerase may result in other mechanisms of telomerase activation including amplifications, rearrangements, and TERC promoter mutations [32]. In vitro coexistence of telomerase and ALT was demonstrated by reconstituting telomerase in the ALT positive GM847 cell line upon hTERT transfection [33]. Persistence of heterogeneous telomeres and APBs suggest that ALT activity was maintained. However, somatic cell hybrids of ALT positive GM847 and telomerase positive GM639 abolished hallmarks of ALT and were telomerase positive. Therefore, there exists an unknown ALT repressor in telomerase positive cells which is active in a few cells even on telomerase knockout, but it is unlikely to be telomerase [33].

## 2. Main Body

### 2.1. Timeline of Early ALT Discoveries

The ALT pathway was first identified in a EST1 negative *Saccharomyces. cerevisiae* mutant in 1993. Most cells which lacked EST1, a gene which encodes a telomerase RNA-associated protein, lost the ability to replicate by telomerase and entered senescence and cell death. However, some survivors spontaneously developed a telomerase-independent maintenance mechanism. *S. cerevisiae* survivors exhibited tandem arrays consisting of both telomeric and subtelomeric DNA sequences which suggested amplification by homologous recombination between distance telomeric and subtelomeric DNA [34]. However, *Saccharomyces pombe*, *Kluyveromyces lactis* and *Ustilago maydis* survivors only extended telomeric DNA sequences [35,36,37]. Untransformed mouse embryonic fibroblasts (MEFs) were generated by extensive passaging of TERC knockout mutants [38]. In 2003, ALT neoplastic transformed mice cells were developed by Chang et al. [39]. Telomerase negative *Caenorhabditis elegans* were first proposed as a useful ALT model based on the observation of heterogeneous telomere lengths and telomeric circles [40]. As recently as 2015, it have been used to identify internal genomic regions which are necessary for telomere duplication [41]. The ALT pathway was first characterized in human cell lines in 1994 when Kim et al. identified two telomerase-negative SV40-immortalized fibroblasts (SW26 and SW13 [42]) using a novel telomerase activity assay [43]. The role of the ALT pathway in a subset of human cancer cell lines and tumors was further investigated in melanomas, osteosarcomas (including SAOS2 and U2OS), and carcinomas of the breast, ovary, lung and adrenal cortex [44,45]. Human ALT cancers exhibit unique biomarkers. In 1999, Yeager et al. identified ALT specific PML bodies which facilitated co-localization of telomeric DNA and telomere binding proteins involved in recombination such as TRF1/2, RAD51, and RAD52. Telomerase negative cells exhibited ALT associated PML bodies (APBs) during immortalization but not wild-type or telomerase positive cells [25]. In 2009, Henson et al. developed a C-circle assay to detect extrachromosomal DNA in ALT cancers. C-circles were detected in the blood of ALT positive osteosarcoma patients. Since then, the C-circle assay is one of the main biomarkers of ALT [46].

In their original 1993 study, the basic mechanism for the ALT pathway was defined by Lundblad and Blackburn who identified two distinct ALT pathways in yeast survivors. The Rad51 dependent type I mechanism is more common in yeast. The Rad52 dependent type II mechanism results in heterogeneous telomeres which are more common in humans. The authors proposed that the Rad52 type II pathway in *S. cerevisiae* survivors required multiple rounds of telomere recombination [34]. In 1999, Teng et al. proposed that critically short telomeres lost telomere binding proteins and invaded a long telomere template strand to initiate telomere recombination [47]. Dunham et al. first demonstrated that ALT cell lines utilize inter-telomeric recombination. In the 2000 study, plasmid tags in telomeric DNA were copied from telomere to telomere in immortalized humans cells [7].

**Figure 1:**
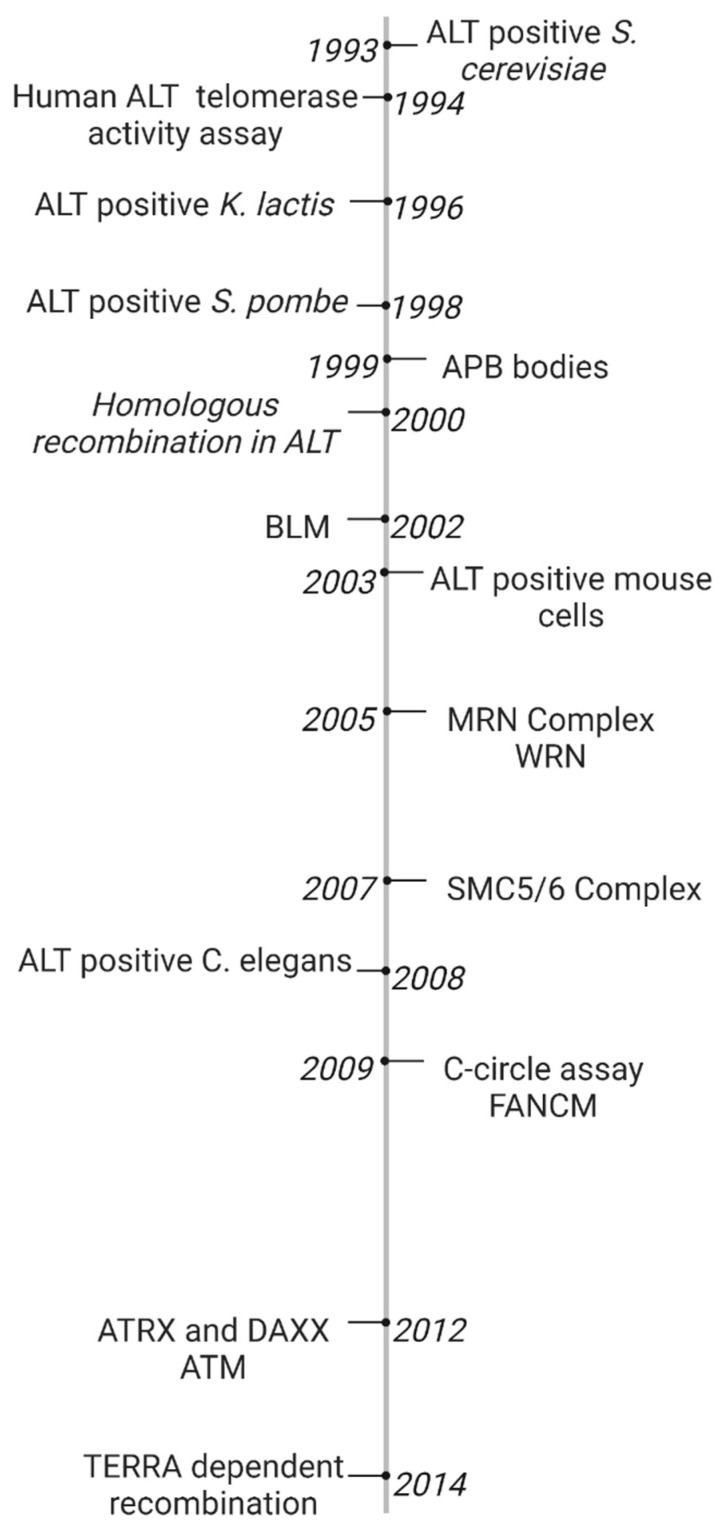
Timeline of ALT Discoveries. The ALT pathway was first identified in *S. Cerevisiae* [26]. A novel telomerase activity assay was used to identify the ALT pathway in human fibroblasts [23]. ALT was later discovered in several model organisms including *K. lactis*, *S. pombe*, mouse embryonic fibroblasts, and *C. elegans* [27,28,30,31]. ALT-associated PML Bodies were the first discovered hallmark of ALT cancer [25]. Extrachromosomal c-rich telomere circles were discovered in ALT cells using a novel C-circle assay [36]. One early model for the ALT mechanism hypothesized that critically short telomeres invaded a long telomere template strand to initiate telomere recombination [7,47]. ALT activation was found to be dependent on TERRA transcription [45]. Many proteins have been linked to telomere recombination, including BLM and WRN helicases [41,42], MRN and SMC5/6 complexes [39], and ATRX and DAXX chromatin remodelers [43,44], and FANCM DNA damage response protein [40].

Since then, many molecular targets have been implicated in telomere recombination. Recombination proteins include the MRN complex, the SMC5/6 complex, and FANCM [48,49,50]. BLM and WRN helicases were found to facilitate telomere recombination [51,52]. Unlike BLM deficiency which promotes recombination at interstitial regions, loss of WRN promotes telomere specific recombination. WRN and TERC double knockout mouse mutants elevate telomere sister chromatid exchanges and activate ALT [52]. DNA damage response proteins involved in ALT activation include ATM, discovered in Atm-deficient mouse cells [53]. Additionally, loss of chromatin remodelers ATRX and DAXX are implicated in ALT activation [54]. The ALT recombinogenic potential is also dependent on telomere repeat-containing RNA (TERRA) transcription. In 2014, Arora et al. demonstrated that TERRA regulates the recombination activity of ALT telomeres by hybridizing with the telomeric C rich sequence. TERRA is regulated by RNaseH1, an RNA endonuclease which associates to telomeres in ALT positive cancers but not telomerase positive cells [55]. The ALT discoveries presented are summarized in chronological order in Figure 1.

### 2.2. ALT Cancer Hallmarks

ALT cancer cells can divide indefinitely (immortal cells) and exhibit break-induced repair (BIR), resulting in several biomarkers that can be used to identify ALT-positive cells. The ALT recombination mechanism results in heterogenous telomere lengths. ALT positive cell lines also exhibit high levels of extrachromosomal telomeric sequences. Circular cytosine-rich telomeric DNA (C-circles) or guanine-rich telomeric DNA (G-circles), usually <1 kb in length, correlate with ALT activity and accumulate in the nucleus [46,56,57]. C-circles are 750 times more common in ALT positive cells compared to normal and telomerase positive cells. C-circle levels are also detectable in blood samples and may be the most useful biomarker for diagnostic tests. C-circles appear to be a nonfunctional byproduct of ALT activity but a more detailed understanding of the formation of C-circles are required to contribute to a more complete model of the ALT mechanism. We speculate that DSBs produced by replication stress in telomeric DNA may create telomere fragments which self-ligate. C-circles which are 100 times more common than G-circles may result from nucleolytic degradation of the G-rich strand of T-circles. Both ALT positive and telomerase positive cancers exhibit T-circles which may be the result of T-loop fragments resolved by recombination enzymes [58]. Extrachromosomal DNA circles accumulate in ALT-associated PML bodies [57].

ALT-associated promyelocytic leukemia nuclear bodies (APBs) comprise one prominent indicator of active ALT activity [25]. The APB matrix is represented by a circular, hollow, membrane-less nuclear structure ranging from 50–100 nm in diameter that is formed primarily from the structural components of PML and SP100 protein [59]. These structures are held together through SUMO-SIM interactions, which are defined primarily as the intramolecular interactions between small ubiquitin-related modifications (SUMO) and SUMO interacting motifs (SIM) [59]. To this complex, telomeric DNA, related proteins, and DNA damage factors are recruited. PML depletion eliminates ALT telomere clustering and synthesis, suggesting that APBs are the location where homologous recombination (HR) occurs to maintain telomere length [60]. Assuming the lack of an additional mechanism to recruit the BTR (Blooms syndrome helicase, topoisomerase IIIa, and RM1/2) complex to telomere ends, APBs are essential to ALT activity [60]. Tethering telomeres to SUMO-SIM fusion proteins and overexpression of BLM helicase results in telomere synthesis and C-circle generation, hallmarks of ALT activity [61]. Loss of the replication stress response proteins FANCM, FANCD2, and SMARCAL1 increases APB formation suggesting that MMS21-mediated SUMOylation of shelterin complex proteins trigger APB formation [49,62,63,64,65].

It has been established that the ALT mechanism relies on recombination between telomere ends and either non-sister chromatids or extrachromosomal sequences. In a previous study, a tag on a single telomere was copied onto other chromosomes ends in ALT positive cell lines but not telomerase positive cells [7]. Additionally, some ALT positive cells exhibit patterns of non-canonical telomere repeats, variants of TTAGGG tandem arrays, suggesting recombination with subtelomeric or other genomic sequences, and possibly extrachromosomal telomere circles [66]. Therefore, ALT positive cancers exhibit increased levels of sister chromatid exchange compared to normal and telomerase positive cells.

Telomeric insertions have been observed across the genome in ALT positive cells. Some spontaneous and experimentally induced DSBs are repaired by insertion of 50–1000-bp sequences derived from distant regions of the genome [67]. RNA transcribed from distant regions of the genome are the primary template sequences for DNA inserted into the genome [67]. However, the mechanism for this mutagenic form of DSB repair remains unclear.

TERRA (Telomeric Repeat-Containing RNA) is RNA transcribed from the telomeres and hybridizes with the C-rich telomeric strand to form RNA/DNA hybrid sequences (R-loops) [55,68]. These R-loops induce recombination events between the ends of chromosomes that elongate telomeres up to >50 kb [55,56,69]. Inhibiting TERRA transcription alleviates ALT activity [70]. This suggests that TERRA is a major trigger of ALT [70]. Additionally, TERRA R-loops form barriers to replication suggesting ALT recombination may be triggered by replication stress [55].

### 2.3. Replication Stress

**Figure 2:**
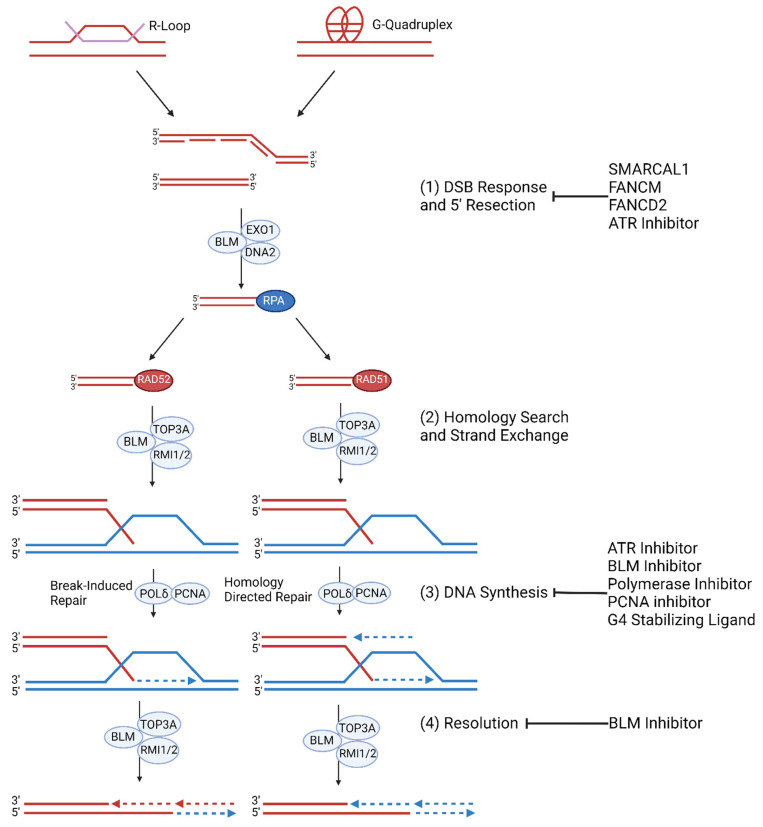
ALT Replication Stress and Molecular Mechanism. (1) Replication stress at telomeres is regulated by SMARCAL1 and FANCM and FANCD2. ALT cells are prone to replication stress which leads to spontaneous DNA synthesis processes. G-quadruplexes and R-loop formation at telomeres trigger replication stress which result in stalled or collapsed replication forks [74,75]. If the replication fork is not reinitiated during S/G2 phase, then mitotic DNA synthesis (MiDAS) of telomeres can occur. (2) The collapsed replication fork may be repaired by RAD52 mediated BIR or RAD51 mediated HR. (3) Telomeric MiDAS-mediated re-initiation leads to conservative DNA synthesis mediated by the BIR pathway when damaged sequences share homology with template DNA. HR- mediated re-initiation leads to semi-conservative DNA synthesis [76]. (4) Extended telomere ends are resolved by BLM [77,78].

ALT cancers exhibit elevated levels of genomic instability and replication stress, but ALT-specific causes of telomeric replication stress are not fully understood. Aberrations in telomeric nucleoprotein structures, including heterochromatin nucleosomes, shelterin complexes, R-loops, and G-quadruplexes may contribute to ALT-specific replication stress (Figure 2) [55,71,72,73].

Telomere heterochromatin may be regulated by a number of ALT-specific epigenetic regulators. Telomere heterochromatin decompaction appears to be a necessary but not sufficient condition for ALT activation via recombination and replication stress. ATRX (α-thalassemia/mental retardation syndrome X-linked) and its binding partner DAXX (death domain-associated protein 6) are tumor suppressing histone chaperones that promote histone H3.3 deposition and remodeling at telomeric regions. ATRX suppresses hallmarks of ALT activity such as the formation of APBs and C-circles [71,79]. Conversely, the loss of either ATRX or DAXX leads to telomeric chromatin decompaction and increased replication stress which promotes HR at the telomeres and may promote ALT activity [71,79,80,81,82,83]. ATRX and DAXX inactivation mutations highly correlate (*p* < 0.008 for each gene) with ALT activity in a variety of tumors including glioblastomas, oligodendrogliomas, medulloblastomas, and pancreatic neuroendocrine tumors [84]. Studies show that loss of ATRX also results in TERRA upregulation and G-quadruplex accumulation at telomeres [54,85]. Therefore, loss of ATRX and DAXX may be important in the initiation or maintenance of ALT replication.

ATRX regulates telomere DSB repair through two pathways involving sister chromatid cohesion or DAXX. ATRX promotes telomere cohesion via the canonical cohesion complex (SMC1-SMC3-RAD21-SA1/2) which is necessary for sister chromatid pairing during mitosis and interphase. Pairing of sister chromatids promotes DSB repair via a sister chromatid template as opposed to a homologous chromosome and prevents unequal sister chromatid recombination, interchromosomal HDR, and joining of distal ends [86,87,88,89,90,91,92]. ATRX deletion in mouse cells promotes defects in telomere cohesion, nonallelic telomere interactions, and homology directed repair (HDR) [93]. Persistent telomere cohesion during mitosis may promote T-SCEs during ALT [94], but the mechanism remains unclear since cohesion usually suppresses break-induced replication (BIR). ATRX also regulates telomere DSB repair through DAXX-dependent pathway. ATRX deletion in DAXX-deficient mouse cells promotes telomere damage, APB formation, and T-SCEs [93]. Therefore, defects in both telomere cohesion and DAXX-dependent function are necessary for telomere DSB repair associated with ALT-specific ATRX deletion [93]. However, some ALT cancers do not exhibit ATRX or DAXX mutations, so they are not essential to ALT cancer activation [95,96,97,98].

Loss of ATRX/DAXX may be associated with telomere insertions—fragments of tandem arrays inserted into non-telomeric regions in a subset of cancers. Some ATRX/DAXX deficient ALT-positive cancers expressed telomere insertions and telomere variant repeats. Longer telomeres correlate with higher telomere insertion event frequency [99]. Additionally, one study found that TERRA was transcribed from these telomere insertions [99]. Another possibility is that telomere insertions occur with ATRX/DAXX mutations regardless of telomere maintenance mechanisms [100]. Thus, the ATRX/DAXX-dependent mechanisms, rather than the ALT mechanism, may be linked to telomere insertions. However, it remains unclear whether there is a relationship between ALT cancers and telomere insertions since the methods for analyzing ATRX/DAXX mutations in ALT cancer datasets is lacking [101].

The nuclear receptor NR2C/F may promote ALT activity by recruiting NuRD (nucleosome remodeling and histone deacetylase) and ZNF827 (zinc finger protein 827) which deacetylate histone H3.3 and promote shelterin loss [102,103]. The unprotected telomeric strand then triggers homologous recombination (HR), a DSB repair pathway, and the remodeled telomeric strand may then promote ALT propagation [102,103]. Additionally, NR2C/F is linked to telomere insertions. NR2C/F binds to telomere variant repeats (GGGTCA) which are elevated in ALT cancers. A small subset of genomic NR2C/F binding sites can interact with telomeric repeats and serve as telomere insertion sites. These telomere fragile sites are prone to DSBs, and the unprotected ends are fused to other chromosome ends via non-homologous end joining. Fused chromosomes may be separated during mitosis, promoting more chromosome deletions, amplifications, breaks, and translocation events [104]. NR2C/F recruit telomeric chromatin which promotes telomere proximity that is necessary for recombination and thus ALT activity. However, NR2C/F may also drive telomere insertions by tethering NR2C/F binding sites to non-telomeric NR2C/F chromatin binding sites, resulting in recombination and insertion of telomeric variant repeats. NR2C/F accumulates in ALT-positive cells and positively correlates with increased telomeric rearrangements. Thus, NR2C/F serves to prevent telomere rearrangements and fusions by maintaining telomere integrity [105].

In normal cells, replication stress halts proliferation and promotes replication stress responses. FANCM (Fanconi anemia complementation group M) is a major suppressor of replication stress inducers such as R-loops. FANCM-deficient yeast cells accumulate R-loops, and ATPase inactive FANCM mutations in yeast fail to resolve R-loops, resulting in elevated levels of DSBs and ALT activity [18,106,107]. This suggests that FANCM interacts with FAAP24 through its ATPase domain to unwind R-loops and resolve stalled replication forks [106]. FANCM is also a major regulator of interstrand crosslink repair through its translocase activity. The FANCM-FAAP24-MHF1/2 complex recruits the FA core complex [108]. DNA lesions trigger FA to monoubiquitinate FANCD2 which localizes to BRCA1/2 and promotes HDR [108]. However, mutations in FANCM fail to suppress FANCD2 monoubiquitination, resulting in HDR. FANCM also interacts with PCNA (proliferating cell nuclear antigen) to remodel arrested replication forks without FA [109,110]. Similarly, SMARCAL1 (SWI/SNF-related matrix-associated actin-dependent regulator of chromatin subfamily A-like protein 1) is an ATP-dependent DNA-annealing helicase which promotes replication fork reversal and re-initiation [111,112,113]. Lesions and barriers in DNA hinder replication machinery, resulting in stalled replication forks. Unrepaired DNA lesions may give rise to DSBs and trigger DSB repair mechanisms and HDR-dependent ALT activity [18,114,115,116].

### 2.4. ALT Molecular Mechanism

ALT HDR is a Break-Induced Replication (BIR) pathway during the G2 and M cell cycle phases [76,117]. DSBs trigger ataxia telangiectasia mutated (ATM)-dependent checkpoint response which recruits BRCA1 complexes or 53BP1 complexes to the lesion. CTIP and MRN (MRE11-RAD50-NBS1) recruit BRCA1 to block 53BP1 from interfering with short range resection—generation of ssDNA overhangs. Exonuclease 1 executes long-range 5′−3′ resection alongside BLM (Bloom syndrome helicase) which unwinds DNA for DNA2 endonuclease. The ssDNA overhangs are filled with the ssDNA-binding factor Replication Protein A (RPA) [77,118]. Experimental BLM overexpression increases RPA at telomeres suggesting that BLM is essential for resection. RPA may then be replaced by RAD51 or RAD52 indicating a RAD51-dependent pathway and RAD52-dependent pathway. BRCA1 and BRCA2 promote recombination by mediating the exchange of RPA and RAD52 recombinase [77]. However, RAD52 depletion and inhibition does not affect C-circle levels suggesting an alternative RAD51 dependent pathway [119]. TERRA contributes to the decision to activate the RAD51 dependent pathway. A previous study showed that TERRA promotes ALT telomere synthesis at APBs via R-loops [55]. In RAD52 knockout conditions, TERRA maintains its ability to form R-loops. TERRA and TRF2 co-localize to APBs in both RAD52 positive and knockout conditions indicating that TERRA localization is independent of RAD52 [120]. Moreover, TERRA knockout or knockdown significantly reduces C-circle levels and telomere synthesis at APBs in RAD52 knockdown cells suggesting that TERRA is essential for RAD51 dependent ALT activity [120]. In RAD51 dependent BIR, the TERRA R-loop promotes R-loop and G-quadruplex formation which allows for the nucleoprotein overhang to replace TERRA, resulting in a transformation from an R-loop to a D-loop [120]. Human cancers rely on the RAD52 dependent pathway. Experimental RAD52 depletion and inhibition decreases telomeric DNA replication [76,119]. RAD51 deletion increases telomeric DNA synthesis and C-circle levels but does not affect BIR or telomere synthesis in APBs [46,119]. In RAD52 dependent BIR, the nucleoprotein 3′ overhang undergoes homology directed search and strand invasion to form a D-loop. In both pathways, the BTR complex (BLM helicase-Topoisomerase 3α-RMI1/2) is recruited to the D-loop in order to unwind the DNA. PCNA, RFC (replication factor C), and polymerase δ replisome elongates the hybridized 3′ overhang [119,121]. FANCM interacts with BTR to enable branch migration of the D-loop which may then be resolved by BTR resulting in extended telomeric DNA and a crossover event mediated by the SMX endonuclease complex (SLX1–SLX4, MUS81–EME1 and XPF–ERCC1) [18,77,78,118]. FANCM, FAAP24, or MHF1/2 depletion increases RPA, BLM, and BRCA1 localization to telomeric DNA indicating increased replication stress. siRNA-depleted FANCM also increases phosphorylated RPA and 53BP1 levels at telomeric DNA [18,114]. Co-depletion of FANCM and BLM or BRCA1 significantly decreases ALT cell viability [122]. BLM overexpression results in increased telomere synthesis, APBs, and C-circles, while BLM depletion results in the opposite effects [51]. SLX4 overexpression also results in decreased telomere synthesis, APBs, and C-circles implicating that SLX4 and BLM antagonize each other [123,124].

### 2.5. APB Formation and Telomere Instability

Given that APBs are essential to ALT activity, PML protein presents an intuitive molecular target since it is an important structural component of APBs. PML-IV tethering to telomeric regions is an important trigger for ALT activity [125]. Furthermore, APBs are exclusively present in ALT positive cells, indicating the importance of targeting APBs to elevate treatment toxicity particularly for ALT cells [25]. SP100 represents an additional constituent of APBs that can be manipulated to inhibit ALT activity. Experimental overexpression of SP100 leads to decreased formation of APBs in ALT cells as well as reduced ALT activity as demonstrated through telomere shortening and the loss of heterogeneous telomere length that is commonly observed with the ALT phenotype [48]. Given the role of MMS21-mediated SUMOylation of shelterin complex proteins in APB formation, the SUMO-SIM interaction is also an appealing target for ALT-specific therapy [62,63,64,65]. Experimental suppression of MMS21, a SUMO ligase responsible for SMC5/6 complex maintenance, results in APB disruption and telomere shortening [49]. Telomere shortening subsequently leads to loss of immortalization and triggers senescence in MMS21-suppressed cells, suggesting that interfering with APB formation may effectively disrupt ALT activity. Additionally, TRF1/2 SUMOylation, a critical step for APB formation involved in telomere heterochromatin regulation, suggests that SUMOylation inhibition may be a viable ALT-specific therapeutic target [49]. In ALT-positive cells, depletion of P300/CBP-associated factor (PCAF) lysine acetyltransferase is also effective at limiting APB formation and ALT activity. Anacardic acid is an effective inhibitor of PCAF, reducing both APB formation and the longevity of the ALT cell lines TG20 and SAOS2. The use of Anacardic acid sensitizes ALT cancers to treatment by radiotherapy [126]. Generally, elevating the interactions of telomeres with the nuclear envelope also decreases APB formation using the experimental development of a RAP1-SUN1 fusion protein [127].

In addition to targeting the structural components of APBs, therapeutic strategies alternatively involve interfering with shelterin protein complex interactions which occur at APBs. USP7, POT1, and ubiquitin ligase interactions rely on APB formation to co-localize which further supports APB-targeting therapeutics [128]. POT1 is critical for shelterin complex formation allowing for the formation of T-loops and thus preventing telomere instability and cell death. USP7 (ubiquitin-specific-processing protease 7) is a proteasome for POT1 and has deubiquitinase activity for POT1 ubiquitin ligases [128]. USP7 is inhibited in ALT-positive cells by TSPYL5 (testis specific Y-encoded-like protein 5) which normally resides in APBs. However, therapeutic suppression of TSPYL5 would result in the derepression of USP7. Then USP7 would co-localize with POT1 within APBs and deubiquitinate POT1 ubiquitin ligases, resulting in POT1 degradation by USP7 proteasomal activity [128]. Thus, TSPYL5 suppression may be an ALT-specific treatment. Additionally, re-expression of a wild-type form of the shelterin component RAP1 in combination with the wild-type version of XRCC1 (a non-homologous DNA end joining repair factor) blocks phenotypic characteristics of the ALT pathway [129].

Furthermore, the shelterin components TRF1 and TRF2, which act as telomere-binding proteins, have been implicated in the formation of APBs and thus the maintenance of the ALT mechanism. The loss of TRF1 and TRF2 results in DNA damage and cell death [130]. Mutation at T271 on the TRF1 protein can limit APB formation, and Cdk-dependent phosphorylation of TRF1 at T371 is essential for TRF1 recruitment to APBs and subsequent APB formation [131]. Also, suppressing TRF1 in mouse cells reduces glioblastoma and lung cancer progression without affecting normal cell viability or tissue health, while inhibiting TRF1 with ETP47228 and ETP47037 prevented TRF1 binding to telomeres and similarly halted cancer progression [132,133,134]. Additionally, kinase inhibitors targeting ERK2, BRAF, mTOR, or AKT may inhibit TRF1 stabilization, resulting in TRF1 suppression and a decrease in ALT activity [132,133,134,135]. TRF2 suppression also interferes with telomere maintenance and T-loop formation, a critical regulatory mechanism in ALT cancers. TRF2 recruits APOLLO exonuclease to resect the 5′ end, leading to the creation of a 3′ end overhang. Without the resulting 3′ ssDNA, telomere ends cannot invade the proximal tandem repeats, and the unprotected telomere ends may trigger DSB-related responses [136,137]. Therefore, the TRF2 and APOLLO exonuclease interaction is a potential target for ALT cancer therapeutics. Additionally, TRF2 suppression has also been linked to HDR protein suppression. Depletion of TRF2 results in SLX4 de-repression and decreased ALT HDR [138,139,140]. Currently, PARP inhibitors are used with BRCA1 or BRCA2-deficient breast and ovarian cancers as DNA damage response suppressors, promoting replication stress and DNA damage [129,141,142,143,144]. Therefore, PARP inhibitors may be promising drug candidates for ALT cancers as well.

### 2.6. Homologous Recombination and Telomeric MiDAS

Distinctive molecular targets can also be identified through independent analysis of the two mechanisms that have been proposed for the ALT pathway based on previous studies with *Saccharomyces cerevisiae*. The type I mechanism corresponding to RAD51-dependent homologous recombination (HR) implicates recombination proteins as useful therapeutic development approaches, while the type II mechanism corresponding to RAD52-dependent BIR implicates molecular targets involved in telomeric MiDAS (mitotic DNA synthesis) [76,145].

The type I pathway relies on RAD51 to maintain ALT activity thus implicating its utility for therapeutic development. RAD51 is primarily involved in HR as it occurs to develop the early precursors in the ALT pathway [146]. RAD51, as well as DMC1, are essential in DNA strand exchange. Interfering with the associated HOP2-MND1 heterodimer required for the appropriate recombinase activity could potentially inhibit HR within ALT cancers [147]. RAD51AP1 may also have a role in mediating the success of both type I and II survivor pathways, with its depletion increasing telomere dysfunction and fragmentation. Yet, inhibition of RAD51AP1 activity does not appear to comprise an effective therapeutic strategy given subsequent activation of ULK1-ATG7-dependent autophagy [148].

The MRN complex, with its RAD50, MRE11, and NBS1 components, also participates in DSB repair through HR. In ALT-negative cells, inhibiting the production of NBS1 results in less frequent sister chromatid exchanges as well as gene conversion [149]. Given the reliance of the type I mechanism on HR, depleting NBS1 in ALT cells results in telomere shortening and the reduction in APB prevalence [150]. This same effect is not observed in telomerase positive cells with NBS1 depletion [150]. This finding coincides with the inhibition of the ALT pathway that results from the overexpression of SP100. SP100 can sequester the MRN complex away from APBs resulting in telomere shortening, decreased telomere heterogeneity, and a reduction in the number of APBs [48]. Mirin has been identified as a small molecule inhibitor of the MRN complex that prevents MRN-dependent ATM signaling, reduces Mre11-related exonuclease activity, abolishes the G2/M checkpoint, and decreases homology-dependent repair [151]. Future therapeutics could target NBS1 for further success in MRN complex inhibition [150].

ATM kinase plays an additional signaling and protein recruitment role in DSB repair via HR in ALT cells and thus poses another target for inhibiting ALT activity [152,153]. ATM activation is a current indicator of resistance to chemotherapy agents temozolomide and irinotecan in ALT neuroblastoma cells; thus, inhibition of Ataxia telangiectasia mutated (ATM) could sensitize these tumors to treatment [153]. An additional study points to the activation of ATM as a sign of ALT cancer’s increased susceptibility to the reactivation of p53 by the drug APR-246 [154]. ATM and rad3-related (ATR) kinase is also involved in DNA damage responses and thus maintains the stability of the HR pathway. Others suggest that ATR inhibitors are effective against ALT cancers [155,156].

Additional recombination-specific factors can also provide potential targets for therapeutic development. For example, the SMC5/6 complex is essential in ALT cells to maintain the structural integrity of chromosomes, participate in DDR pathways, and thus ensure the effective progression of HR. The inhibition of the SMC5/6 complex prevents telomere HR which results in the shortening of the cell’s telomeres and the cell’s eventual entrance into senescence [49]. RPA, BRCA1, BLM, FANCM, WRN, and the SLX1/SLX4/ERCC4 complex comprise additional targets with the inhibition of RPA, BLM, WRN, and FANCM (particularly when co-depleted with BLM) and the overexpression of BRCA1 and SLX1/SLX4/ERCC4 being most promising [51,122,124,125,157,158,159,160,161].

Pol δ is associated with conservative DNA replication, characteristic of the type II RAD52 dependent pathway, providing an additional molecular target to limit telomere elongation [161,162]. Depletion of POLD3 and POLD4, two subunits of Pol δ, decreases viability of cells that overexpress cyclin E by preventing their entry into cell cycle S phase [76]. In addition to reducing recombination events related to BIR [121], POLD3 is particularly relevant for MiDAS (Mitotic DNA Synthesis) [103]. The mechanism for the telomeric MiDAS that occurs in type II survivors is derived from CSF-MiDAS [61]. In CFS-MiDAS, MUS81-EME1 and XPF-ERCC1 are required to function in association with POLD3 and the SLX4 protein as structure-specific endonucleases, and the depletion of either ERCC and/or MUS81-EME1 results in mitotic catastrophe and cell death [163,164,165,166]. RAD52 depletion has similar consequences to the depletion of MUS81 and POLD3 and is associated with blocked progression of CSF-MiDAS. Similar to CFS-MiDAS, telomeric MiDAS requires SLX4 and RAD52 although MUS81 is not essential [123]. Furthermore, the CFS-MiDAS component XPF encourages ALT activity through break-induced synthesis given its activation of DDR pathways that result from the formation of telomeric R-loops [167]. Targeting SLX4, RAD52, and XPF could thus limit ALT activity through reducing telomeric MiDAS activity. Furthermore, while the expression of the TIMELESS/TIPIN complex limits telomeric MiDAS, the activity of the SMC5/6 complex encourages telomeric MiDAS, further supporting the role inhibition of SMC5/6 in the development of a therapeutic approach for ALT cancers [76].

Post-MiDAS telomere replication may be another therapeutic target. ALT-positive telomere replication promotes heritable ssDNA telomeric lesions. Replication stress gives rise to endogenous DNA damage and results in unfinished replication as well as RPA-marked ssDNA lesions in daughter cells. Telomeres are one type of fragile DNA due to its highly repetitive and heterochromatic nature [168]. Some under-replicated regions and lesions from the G2/S cell cycle phase can evade checkpoint responses and be replicated during mitosis. MiDAS is a specialized BIR pathway outside of S-phase which continues replication. If replication remains incomplete, DNA lesions are inherited by the daughter cells in the G1 phase. These telomere lesions may trigger ALT telomere maintenance mechanisms [169]. RPA-marked telomeres are detectable in G1 cells which is indicative of ssDNA lesions despite the fact that resection is blocked by 53BP1 and HDR is inhibited in the G1 phase. RPA-marked telomeres also co-localize to APBs implicating replication stress in ALT activity [169]. Thus, ALT-positive cells are susceptible to replication stress-induced heritable ssDNA lesions in G1 daughter cells. RPA protects ssDNA lesions and can be implicated in further telomere replication. Given that RAD52 is involved in BIR, knockdown of the MiDAS-associated RAD52 in G1 cells reduces RPA-marked telomere lesions [169]. Telomere replication at RPA and RAD52 marked lesions occurs in the G1 phase in a process analogous to MiDAS called post-MiDAS. Given that replication stress is a hallmark of ALT-positive cancers and RPA protects inherited ssDNA lesions, therapeutic strategies targeting post-MiDAS in G1 phase are worthy of investigation [169].

### 2.7. Hyperactive ALT Pathway

While some types of ALT therapeutics can target functional molecular mechanisms to halt the progression of the ALT pathway, another type of ALT therapeutic creates a hyperactive ALT phenotype with increased replication stress and the subsequent acceleration of DNA damage [170]. One effective way to generate these therapeutics is to assess regulators of ALT activity. For example, one limiting factor of ALT activity can be found in cell cycle regulation associated with the activity of WEE1 and PKMYT1 proteins. The WEE1 protein phosphorylates the CDK1/Cyclin B1 and the CDK2/Cyclin A/E complexes at the Tyr15 position, ultimately preventing the accumulation of DNA damage during the S phase and chromosome pulverization during the G2/M phase. Similarly, the PKMYT1 phosphorylates the Tyr15 and Thr14 positions of the CDK1/Cyclin B1 complex [171]. Targeting the WEE1 protein with inhibitors such as MK-1775 and the PKMYT1 protein with inhibitors such as RP-6306 is particularly promising given the heightened sensitivity of cells with ATRX-deficiencies to WEE1 inhibitors [167,172].

Aside from the WEE1 and PKMYT1 proteins, another targetable component involved in mitigating the consequences of replication stress on ALT cells includes SMARCAL1. Although not associated with significantly greater recombination rates and telomere lengths typical of ALT cells, the depletion of SMARCAL1 correlates with an increase in C-circle production [112]. In the absence of SMARCAL1, increased telomere dysfunction is observed in the form of DNA DSBs and chromosome fusion [113]. ATM also functions as a replication stress response protein. AZD0156 is an ATM inhibitor with potential anti-neoplastic activity that targets ALT positive neuroblastomas [173,174]. Given its association with replication fork regression and stabilization and the mediation of DSB repair, inhibiting ATR may promote increased replication stress and decreased DSB repair capacity which is lethal for ALT positive cells. Studies show that inhibiting ATR is more toxic to ALT-positive cells compared to telomerase-positive cells [175,176,177,178].

FANCM is another replication stress response protein with therapeutic potential given its activity as an ATPase and DNA translocase as well as its involvement in the resolution of TERRA R-loops and the restarting of stalled replication forks [116,170,174]. FANCM suppresses ALT activity such as telomeric DNA damage and C-circle generation [18]. FANCM depletion thus results in increased replication stress, telomere dysfunction, reduced replicative activity, and reduced cell viability [18,122]. FANCM inhibition is lethal to ALT-positive cells by arresting the cell cycle in the G2/M phase [18,114]. However, FANCM depletion in normal and telomerase-positive cells does not promote ALT activity of induce cell cycle arrest, making FANCM a candidate for ALT cancer therapeutics [18,108]. Given the interaction between FANCM and BTR, the dual inhibition of the FANCM-BTR complex reduces the viability of ALT cells via increased break-induced telomere synthesis [114,179]. Targeting the FANCM-BTR interaction by expression of a peptide corresponding to the MM2 domain of FANCM, which interferes with branch migration of the D-loop, significantly reduces cell viability [114]. The small molecule inhibitor PIP-199 is another FANCM-BTR inhibitor [114,180]. Depletion of FANCD2, related to the FANCM compound through the former’s monoubiquitination, induces a hyperactivation of ALT activity and leads to DNA damage accumulation and cell death [170,181].

Promoting replication stress by stabilizing G-quadraplexes may also reduce ALT cancer viability. G-quadraplex-stabilizing ligands may promote G-quadraplex formation, resulting in DSBs, DNA damage, and cell death [182]. For example, telomestatin not only inhibits telomere synthesis in telomerase-positive cell lines but also destabilizes the shelterin complex formation, resulting in replication stress and cell cycle abrogation [183]. Additionally, the pentacyclic acridine compound RHPS4, pyridostatin, and 2,6-pyridine-dicarboxamide derivatives lead to the generation of APBs and C-circles in ALT-positive cells suggesting telomere replication stress induction and ALT activity promotion [184,185]. An additional strategy includes depleting the helicases such as FANCJ-BLM that would subsequently lead to the stalling of telomere replication [186].

## 3. Conclusions

In this review, we summarized ALT mechanisms of telomere maintenance to provide potential future more specific ALT therapeutic strategies (Table 1). The current ALT framework involves two BIR mechanisms–RAD51 dependent and RAD52 dependent–induced by telomere damage and replication stress. ALT activity can be detected by several hallmarks including C-circles, TERRA, APBs, G-quadruplexes, and a number of replication stress response proteins including FANCM, BLM, and SLX4. RAD51-dependent HR implicates recombination proteins as therapeutic targets such as NBS1, SMC5/6, Pol δ. RAD52-dependent BIR implicates proteins involved in telomeric MiDAS such as RAD52, POL3/4, and SLX4. We discussed new therapeutic strategies that seek to create a hyperactive ALT cell which is sensitive to DNA damage by targeting FANCM, WEE1, PARP, shelterin components, and G-quadruplexes. Another strategy targets APB formation, the site of telomere clustering and synthesis.

While not common, some cancers may switch to ALT when telomerase is inactivated, so therapies to inhibit telomerase and ALT are equally important for cancer therapy [32,76]. A better understanding of the ALT mechanism may identify and lay the foundation for ALT-targeting cancer therapies. In this manner, by targeting molecules that participate in protecting ALT telomeres, forming APBs, or completing homologous recombination, break-induced telomere synthesis, or telomeric MiDAS, the ALT pathway can be manipulated. ALT targeting cancer therapeutics will most likely combine several inhibitors. Redundancy in APB, HR, BIR suppression may be required to inhibit several levels of ALT activity without affecting normal cells.

We suggest that targeting ALT may be a beneficial approach to treating a subset of human cancers because it offers a unique way to exploit tumor dependency on ALT in cancers with poorer prognosis. Advances in ALT studies have begun to elucidate the close relationship between the ALT molecular mechanisms and DNA damage repair pathways—BIR and HDR—which are implicated in dysfunctional and neoplastic cells. However, ALT studies are currently limited to in vitro studies, and the clinical applications of ALT therapies are unproven.

## Figures and Tables

**Table 1:** ALT Therapeutic Targets.

Therapeutic Target	Molecular Mechanism	Relevant Drugs
SP100	MRN sequestering and APB inhibition	
TRF1 (T271)	APB formation	
TRF1 (T371 phosphorylation)	APB formation	
MMS21 SUMO ligase	SMC5/6 complex maintenance interference	
TSPYL5	USP7 repression	
FANCM/FAAP24	Replication stress suppression	
FANCM/BTR	D-loop branch migration	PIP-199
ATRX/DAXX	Chromatin decompaction and telomere cohesion	
NRSC/F	NuRD and ZNF827 recruitment	
SMARCAL1	Replication fork reversal and re-initiation	
FA core complex	FANCD2 monoubiquitination and BRCA1/2 localization	
TRF2	APOLLO exonuclease recruitment and 5′ resection	
TRF2	SLX4 repression	
PARP	BRCA1/2 interaction	
HOP2-MND1 heterodimer	Recombinase activity	
DMC1	DNA strand exchange	
RAD51AP1	Telomere dysfunction and fragmentation	
NBS1	T-SCEs	
MRN	ATM signaling	Mirin
POLD3/4	BIR and CFS-MiDAS	
XPF	DDR pathway activation	
WEE1	CDK phosphorylation	MK-1775
PKMYT1	CDK phosphorylation	RP-6306
ATM	Replication form regression and stabilization	AZD0156

## Data Availability

Utilizing key phrases including, but not limited to, “ALT cancer therapeutics”, “ALT mechanism”, “ALT-associated PML Bodies”, and “telomeric MiDAS”, previous review articles were collected using the Google search engine [181,187,188,189,190,191,192,193,194,195]. These articles were subsequently dissected to find useful sources utilizing the PubMed.gov database from the National Library of Medicine as well as the Columbia University Library System. Sources were evaluated based on their relevance to five subcategories: Telomere Protection, APB Formation, Homologous Recombination, Break-Induced Telomere Synthesis and telomeric MiDAS, and Hyperactive ALT pathways. Regardless of utility, sources were excluded if published before 1997 to ensure that this review encapsulated the most recent developments. Figures (Figure 1 and Figure 2) were created with BioRender.com (20 March 2023).
